# Promotion of flavonoid biosynthesis in leaves and calli of ornamental crabapple (*Malus* sp.) by high carbon to nitrogen ratios

**DOI:** 10.3389/fpls.2015.00673

**Published:** 2015-09-01

**Authors:** Huihua Wan, Jie Zhang, Tingting Song, Ji Tian, Yuncong Yao

**Affiliations:** ^1^Department of Plant Science and Technology, Beijing University of AgricultureBeijing, China; ^2^Key Laboratory of New Technology in Agricultural Application of Beijing, Beijing University of AgricultureBeijing, China

**Keywords:** crabapple, carbon (C), nitrogen (N), flavonoid, anthocyanin

## Abstract

Flavonoids are secondary metabolites that play important roles in plant physiology. Despite numerous studies examined the effects of available carbon (C) or nitrogen (N) on flavonoid biosynthesis, the mechanism of C/N interactive effects on flavonoid metabolism is still unclear. In this study, we analyzed the composition of flavonoids and the expression levels of flavonoid-related genes in leaves and calli of crabapple (*Malus* sp.) cultivars with different leaf colors grown on media with different C/N ratios. Our results show that high C/N ratios induce anthocyanin pigmentation in leaves of the ever-red cultivar ‘Royalty’ and the spring-red cultivar ‘Prairifire,’ as well as in three types of calli derived from the ever-green cultivar ‘Spring Snow,’ but not in the leaves of the ever-green cultivar ‘Flame.’ This phenomenon therefore correlated with anthocyanin content in these different samples. In addition, high C/N ratios in the growth media resulted in an increase in the concentration of flavones and flavonols in the leaves of the three crabapple cultivars. The transcript levels of the general flavonoid pathway genes [from *chalcone synthase (CHS)* to *uridine diphosphat-glucose: flavonoid 3-O-glycosyltransferase* (*UFGT*) and *flavonol synthase* (*FLS*)] increased in response to high C/N ratios, and this in turn was correlated with the concentration of anthocyanins, flavones and flavonols in the leaves and calli. Expression of the late flavonoid/anthocyanin biosynthetic genes, *anthocyanidin synthase (ANS)*, *UFGT* and *FLS* in particular, was more strongly influenced by C/N ratios than other structural genes, and the increased expression of the structural genes under high C/N ratios coincided with a coordinated increase in transcript levels of a MYB transcription factor, *MYB10*. These results are likely to be useful for future generation of plants with an optimized flavonoid/anthocyanin content or desirable organ coloration.

## Introduction

Atmospheric carbon dioxide (CO_2_) concentration is projected to rise to 500–1000 mmol mol^-1^ by the end of this century (Susan, 2007), and this increase will not only accelerate the greenhouse effect but also alter the C and N balance in the global ecosystem (Carlisle et al., 2012; Fulweiler et al., 2014). The availability of CO_2_ is regarded as one of the major limiting factors in photosynthesis, which is the mechanism by which carbohydrates are synthesized for subsequently use as an energy source and to provide C-skeletons for plant metabolism, growth, and development (Broadmeadow and Jackson, 2000; Jaafar et al., 2012; Sun et al., 2012). In addition, to C availability, N is an essential macronutrient and a key factor in regulating plant metabolism and aspects of development, such as root architecture (Zhang and Forde, 1998), leaf formation (Walch-Liu et al., 2000), seed dormancy (Alboresi et al., 2005), and flowering (Stitt et al., 2002). However, cellular C and N metabolism must be tightly coordinated to sustain optimal growth and development in plants and other organisms. Carbohydrates can provide both the energy and the C-skeletons used for N assimilation during amino acid biosynthesis, while amino acids and proteins are the key building blocks for the cell (Coruzzi and Zhou, 2001). Organisms must therefore monitor both the individual status of cellular C and N and as well as the balance between C and N to optimize their metabolism, growth, and development. Despite numerous studies focusing on the biochemical and physiological aspects of C or N metabolism (Tallis et al., 2010; Sun et al., 2012; Fulweiler et al., 2014; Takatani et al., 2014; Marble et al., 2015), there is still a lack of a detailed understanding of the molecular mechanisms underlying the interaction between C and N.

Flavonoids are a subgroup of polyphenols, which can be found in many plant taxa (Andersen and Markham, 2010; Søltoft et al., 2010; Falcone Ferreyra et al., 2012). According to their chemical structures, flavonoids can be classified into six major subgroups: chalcones, flavones, flavonols, flavandiols, anthocyanins, and condensed tannins (or proanthocyanidins). These metabolites have a broad spectrum of action in plants, such as providing flowers and seeds with pigmentation to attract pollinators and seed dispersers, and protecting plants from drought stress, UV radiation, or pathogen infection and reducing the risk of oxidative damage (Winkel-Shirley, 2001; Gould et al., 2002; Jaakola et al., 2014; Mouradov and Spangenberg, 2014). Flavonoids also have a wide range of properties with potential pharmacological value, such as free-radical scavenging, antioxidant function, use as estrogenic supplements, anti-inflammatory characteristics, anticancer potential, and anti-lipid peroxidation (Kang et al., 2003; Liu, 2003; Lila, 2004). Thus, in addition to playing important roles in plant development and environmental adaptation, flavonoids have been shown to be beneficial to human health (Liu, 2003; Koes et al., 2005).

Due to the great biological and agricultural importance and health benefits, the flavonoid biosynthetic pathway has been intensively studied in several plant species (Holton and Cornish, 1995; Winkel-Shirley, 2001; Grotewold, 2006). For example, in *Malus* crabapple, seven structural genes encoding for CHS (Tai et al., 2014), CHI (Geng et al., 2010), F3H (Shen et al., 2012), F3′H (Qin et al., 2013), DFR (Wen et al., 2010), ANS (Tian et al., 2010), and UFGT (Han et al., 2014) have been identified and characterized. The expression of these structural genes is largely regulated at the transcriptional level by the MYB-bHLH-WD40 (MBW) TF complex (Koes et al., 2005; Hichri et al., 2011; Outchkourov et al., 2014; Schwinn et al., 2014; Wang et al., 2014). Previous studies showed that MYB10 played an important role in anthocyanin accumulation during petal or leaf coloration, by positively regulating F3′H and later structural genes in crabapple (Jiang et al., 2014; Tian et al., 2015). Flavonoid biosynthesis is also known to be affected by environmental factors, such as light (Albert et al., 2009; Jaakola et al., 2014), temperature (Xie et al., 2012), water stress (Tattini et al., 2004), environmental pH (Zhang et al., 2014), as well as the phytohormones gibberellins, jasmonate, and abscisic acid (Loreti et al., 2008).

Carbohydrates have been shown to have stimulatory effects on flavonoid synthesis in different organs of several plant species, such as radish (*Raphanus sativus*) hypocotyls (Hara et al., 2003), rose (*Rosa hybrida* ‘Pusa Ajay’) calli (Ram et al., 2011), and *Cleome rosea* calli (Simões et al., 2009). In *Arabidopsis thaliana* seedlings, sucrose specifically induced both higher flavonoid and anthocyanin content and the expression of genes involved in the flavonoid biosynthetic pathway (Solfanelli et al., 2006). Moreover, the TF *MYB75/PAP1* was shown to be essential for the sucrose-mediated expression of *DFR* (Teng et al., 2005). Similarly, sugars have been reported to induce the transcription of genes involved in anthocyanin biosynthesis as well as pigment accumulation in developing corollas of petunia (*Petunia hybrida*), while petunia corollas cultured *in vitro* without sucrose did not show any pigmentation (Weiss, 2000). Finally, recent studies have shown that elevated CO_2_ increased anthocyanin biosynthesis during delayed autumnal senescence (Tallis et al., 2010).

N deficiency has also been shown to strongly induce increased levels of anthocyanins and other flavonoids. For example, in tomato (*Solanum lycopersicum*) leaves, the content of anthocyanins, and particularly petunidin and the flavonol conjugate quercetin-3-*O*-glucoside, consistently increased two- to threefold under N-deficient conditions, and the transcription levels of *CHS* and *DFR* also increased greatly (Bongue-Bartelsman and Phillips, 1995). In seedling and rosette stage plants of *A. thaliana* wild type and *pap1D* mutant, N-deficiency strongly induced the transcription of the TF genes *PAP1/PAP2* (*PRODUCTION OF ANTHOCYANIN PIGMENT 1/2*) and *GL3* (*GLABRA3*), and resulted in the accumulation of both anthocyanins and flavonols (Lea et al., 2007). Further studies showed that *GL3*, but not *EGL3* (ENHANCER OF *GLABRA3*), was necessary for anthocyanin and flavonol accumulation as induced by N depletion in *A. thaliana* rosette stage leaves (Feyissa et al., 2009). It was also found that when grown under N-deficient conditions, transgenic *A. thaliana tt7 (transparent testa7-1)* seedlings expressing an apple *F3*′*H* gene regained red color pigmentation and showed a significant accumulation of both 4′-hydrylated pelargonidin and 3′,4′-hydrylated cyanidin (Han et al., 2010).

*Malus* crabapples belong to the Rosaceae family and are among the most economically important ornamental plants. Crabapples have many desirable ornamental traits and have great value as model plants, since they exhibit excellent stress resistance and are useful for investigating the mechanisms of plant pigmentation, due to the diversity of color in their leaves, flowers and fruits as a consequence of anthocyanins accumulation (Tai et al., 2014; Zhang et al., 2014).

This current study of the plantlets and calli of crabapple cultivars with different leaf colors grown under varied C/N conditions sought to: (i) determine the effects of the C and N nutrient balance on crabapple growth; (ii) establish whether high C/N could be employed to increase flavonoid accumulation in different tissues, identifying and quantifying the flavonoids present in crabapple plantlets and calli; (iii) test whether C/N ratios could regulate the expression of flavonoid biosynthetic and regulatory genes; (iv) reveal the relationship between varied C/N conditions and the expression levels of flavonoid-related genes and the accumulation of flavonoids. We observed that high C/N ratios (both high C/normal N and normal C/low N) resulted in an increase in the transcript levels of genes from the crabapple flavonoid/anthocyanin pathways, most likely via *MYB10* action, and a consequent increase in the accumulation of anthocyanins, flavones, and flavonols.

## Materials and Methods

### Plant Material and Growth Conditions

Four crabapple cultivars (*Malus* sp.) were selected based on their leaf color, including one ever-red leaf cultivar (*Malus* ‘Royalty’) one spring-red leaf cultivar (*Malus* ‘Prairifire’), and two ever-green leaf cultivars (*Malus* ‘Flame’ and *Malus* ‘Spring Snow’). The experimental material was collected from one-year old branches of 5-year old crabapple trees grafted on *Malus* ‘Balenghaitang’ in the Crabapple Germplasm Resources Garden at the Beijing University of Agriculture, Changping District in Beijing.

*In vitro* grown plantlets of four crabapple cultivars were cultured on MS medium (Murashige and Skoog, 1962) with 0.6% agar under controlled environmental conditions, as previously described (Jin et al., 2011). Pyknotic calli, medial calli, and porous calli were induced from leaf explants on MS medium containing 4 mg.L^-1^ 6-BA plus 2 mg.L^-1^ NAA, 0.1 mg.L^-1^ 6-BA plus 2 mg.L^-1^ 2,4-dichlorophenoxy acetic acid (2,4-D) and 0.01 mg.L^-1^ 6-BA plus 2 mg.L^-1^ 2,4-D, respectively. All calli cultures were maintained under the same physical conditions as previously described (Jin et al., 2011), with subculturing at 30-day intervals for pyknotic calli and medial calli, and at 15-day intervals for porous calli. All tissue cultures investigated in this study were preserved at the Tissue Culture Center at the Beijing University of Agriculture.

### C and N Balance Response Assays

Plantlets and calli were grown on MS medium modified with different concentrations of sugars and total N. Sucrose was used as C source. And the concentration of total N was calculated as the sum of NO_3_^-^ and NH_4_^+^. Eight C/N levels were designed as shown in **Table 1**, and 90C/60N was used as a control. After treatments, all samples were collected at 30 day with the exception of porous calli, which were collected at 15 day. Samples were immediately frozen in liquid N and stored at –80°C until further use.

**Table 1:** Treatments of varied C/N ratios.

Treatment	Sucrose concentration (mM)	Total N concentration (mM)	Ammonium nitrate concentration (mM)	Potassium nitrate concentration (mM)
Tr1	30	60	20	20
Tr2	90	60	20	20
Tr3	150	60	20	20
Tr4	210	60	20	20
Tr5	270	60	20	20
Tr6	90	100	40	20
Tr7	90	40	10	20
Tr8	90	20	0	20

### Growth Index Determination

The FW increment was calculated by determining the difference in FW of plantlets or calli at inoculation and the FW when collected at 15 or 30 day. The FWs of all experimental plantlets and calli were measured using an electronic balance, and the height of regenerated shoots was measured using a ruler. The proliferation rate was recorded as the number of new micro-shoots that were produced per subculture.

### Color Analysis

The crabapple leaf color variables of randomly selected leaves from the cultivars used in this study were measured using the CIE L*a*b* scale of the International Commission on Illumination (CIE 1986). The colors of three fresh leaves per plant grown under each C/N level condition were measured using a spectrophotometer CR400 (Konica Minolta, Japan). For each leaf, both the adaxial and abaxial surface were measured at the same mid-point of the leaf under the condition C (330–780 nm, 6774 K) and a viewing angle of 2°. The lightness coefficient ‘L*,’ represents brightness and darkness, the ‘a*’ value represents greeness and redness as the value increases from negative to positive, and the ‘b*’ represents blueness and yellowness. The hue angle h° = tan^-1^(b*/a*) (Lu et al., 2009) was calculated to further characterize leaf color.

### High Performance Liquid Chromatography (HPLC) Analysis

The analysis of flavonoid profiles was carried out using high pressure liquid chromatography (HPLC). Approximately 0.2 g (FW) of each sample was extracted with 10 mL methanol:water:formic acid:trifluoroacetic acid (70:27:2:1; v/v) (Hashimoto et al., 2000). The extract was briefly vortexed and then kept at 4°C in the dark for 72 h with shaking every 6 h. The supernatant was then filtered through sheets of qualitative filter paper, and then through a 0.22 μm Millipore filter (Billerica, MA, USA). The flavonoids were analyzed using a HPLC1100-DAD system (Agilent Technologies, Waldbronn, Germany) with a NUCLEODURH nC18 column (250 mm, 64.66 mm; Pretech Instruments, Sollentuna, Sweden) operating at 25°C. The mobile phase solvent A was water:formic acid:trifluoroacetic acid (97.9:2:0.1; v/v) and the solvent B was water:acetonitrile:formic acid:trifluoroacetic acid (62.9:35:2:0.1; v/v). The column was treated with a gradient solvent system that was designed to separate metabolites (**Table 2**). The flow rate was 0.8 mL/min and the injection volume was 10 μL. Detection was performed at 520 nm for anthocyanin and 350 nm for flavones and flavonols (Revilla and Ryan, 2000). Three biological replicates were analyzed for each sample type.

**Table 2:** Design of the gradient solvent system.

Mobile A (%)	Mobile B (%)	Time/minute
70	30	0
65	35	5
60	40	10
55	45	20
50	50	30
45	55	50
40	60	70
70	30	80

### Quantitative Real-Time PCR Analysis

Total RNA was isolated from frozen samples with TRIzol reagent (Promega, USA). First strand cDNA was synthesized from 1 μg of total RNA using the SMART^TM^ PCR cDNA Synthesis Kit (Clontech, USA) with random hexamer primers (Promega, USA), according the manufacturer’s recommendations. Quantitative real-time PCR (qRT-PCR) was performed using SYBR^®^ Premix Ex TaqTM II (Perfect Real Time) (Takara, Japan) with the CFX96TM Real Time System (Bio-Rad, USA). The PCR amplification conditions were as described by (Shen et al., 2012). The primers were designed with the Primer 5 software and their sequences are listed in **Table 3** (Lalitha, 2000). Relative quantification of specific mRNA levels was performed using the cycle threshold (Ct) 2^-ΔΔCt^ (Livak and Schmittgen, 2001). For all analyses, the signal obtained for each gene of interest was normalized against the signal obtained for the *M. domestica* 18S ribosomal RNA (accession number: DQ341382). Three biological replicates and three technical replicates were analyzed for all samples.

**Table 3:** Primers for quantitative real-time PCR (qRT-PCR) analysis.

Accession number	ID	Sequence (5′–3′)	Product length
JX162681	McMYB10-F	ACGCCACCACAAACGTCGTCG	220 bp
	McMYB10-R	GGCGCATGATCTTGGCGACAGT	
FJ599763	McCHS-F	TGACCGTCGAAGTTCGC	182 bp
	McCHS-R	TTTGTCACACATGCGCTGGA	
FJ817486	McF3H-F	ACGAAGACGAGCGTCCAAAG	233 bp
	McF3H-R	CTCCTCCGATGGCAAAGCAA	
KF481684	McF3′H-F	CGTTGCTGTCGCTCACGGATGA	108 bp
	McF3′H-R	ATGACGTGTCAGTGCCAGCTGTG	
FJ817487	McDFR-F	CCGAGTCCGAATCCGTTTGT	126 bp
	McDFR-R	CCTTCTTCTGATTCGTGGGGT	
FJ817488	McANS-F	CACAGGGGCATGGTGAACAA	202 bp
	McANS-R	TTCACTTGGGGAGCAAAGCC	
KF495603	McUFGT-F	TGGGCGGACACCAATCA	194 bp
	McUFGT-R	ATGTCTCCACCGCACCA	
KF481683	McFLS-F	ACGAGCAACCGGGAATCACAACTG	120 bp
	McFLS-R	CCCAGTTGGAGCTGGCCTCAGTA	
DQ341382	18S RNA-F	GTCACTACCTCCCCGTGTCA	102 bp
	18S RNA-R	GAGCCTGAGAAACGGCTACC	

### Statistical Analysis

All results were analyzed by a one-way analysis of variance (ANOVA) followed by a Duncan’s multiple range test to compare the differences among eight treatments at *P* = 0.05. Pearson correlation coefficients were calculated and a two-tailed test was used to determine significance at the 5 and 1% levels. Microsoft Excel 2003 and DPS 7.05 (Hangzhou, China) were used for all calculations, and OriginPro 8 (OriginLab Corporation, USA) was used for graphing.

## Results

### Carbon and Nitrogen Balance can Affect the Growth of Crabapple

To investigate the influence of the C and N nutrient balance on the growth of crabapple, plantlets of ‘Royalty,’ ‘Prairifire,’ and ‘Flame,’ as well as three types of calli, were grown on modified MS media containing various C/N levels. In all samples, significant differences in growth indicators were observed as a consequence of the various C/N levels.

**FIGURE 1:**
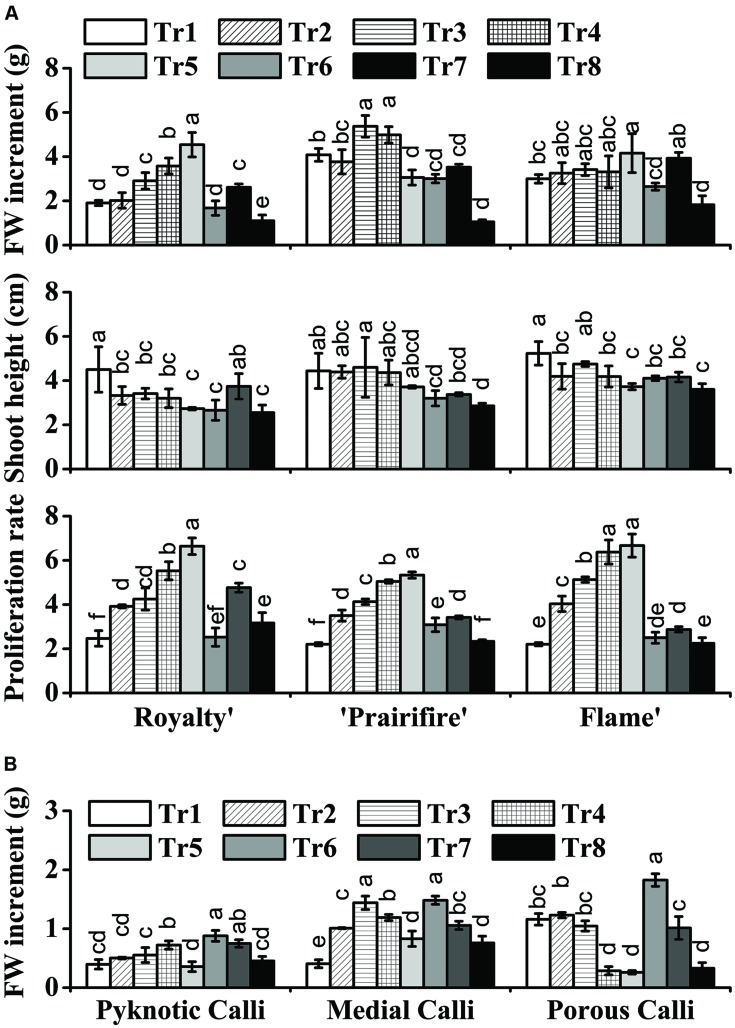
**Effects of C and N nutrient balance on the growth of crabapple *Malus* sp. cultivars. (A)** Effects of C/N ratios on the FW increment, shoot height, and proliferation rate of the plantlets of the ‘Royalty,’ ‘Prairifire,’ and ‘Flame’ cultivars. Plantlets were grown on MS media with 6-BA and NAA, varying C/N levels and harvested on day 30. **(B)** Effects of C/N ratios on the FW increment of the pyknotic calli, medial calli, and porous calli induced from ‘Spring Snow.’ Calli were grown on the MS media with 6-BA, NAA, or 2,4-D, varying the C/N ratios. Pyknotic and medial calli were harvested on day 30, and porous calli on day 15. Tr1–Tr8 correspond to 30C/60N, 90C/60N, 150C/60N, 210C/60N, 270C/60N, 90C/100N, 90C/40N, and 90C/20N, respectively. Error bars correspond to the SEM ± SE of three replicate analyses. Different letters above the bars indicate significantly different values (*P* < 0.05) calculated using one-way analysis of variance (ANOVA) followed by a Duncan’s multiple range test.

With respect to the plantlets, in media with 60N, increasing C from 30 to 270 mM led to an increase in FW for ‘Royalty,’ ‘Prairifire,’ and ‘Flame,’ with the highest FW increment at 270, 150, and 270 mM, respectively (**Figure 1A**). In contrast, the shoot height of the three crabapple cultivars was markedly reduced as C increased (**Figure 1A**). The proliferation rate of ‘Royalty’ increased from 2.47 to 6.64 as C increased from 30 to 270 mM at a constant N level (60 mM), and that of ‘Prairifire’ and ‘Flame’ increased from 2.21 to 5.33, and from 2.21 to 6.67, respectively (**Figure 1A**). When N decreased from 100 to 20 mM while C was kept constant (90 mM), the FW increment, the shoot height and proliferation rates of the plantlets of three cultivars all increased at first and then declined (**Figure 1A**). We concluded that a suitable high C concentration yielded an increase in FW, a reduction in shoot height and amplified the proliferation rate of crabapple plantlets to different degrees under certain N concentrations.

The FW increment of the calli all increased initially, and then decreased as C increased from 30 to 270 mM at a constant N level (60 mM). The pyknotic calli showed the highest FW increase (0.72 g/jar) under 210C/60N, while medial calli and porous calli showed the maximum FW increase under 150C/60N and 90C/60N, respectively (**Figure 1B**). In media with 90C, the biomass accumulation of pyknotic calli fell initially and then rose with N decreased from 100 to 20 mM. The mass of both medial and porous calli declined with N decreased (**Figure 1B**). The three types of calli all exhibited the lowest and the highest biomass accumulation under 90C/20N and 90C/100N, respectively, increasing one- to twofold (**Figure 1B**). This suggests that N availability influences calli growth.

### High C/N Induces Anthocyanin Accumulation in Plantlets

**FIGURE 2:**
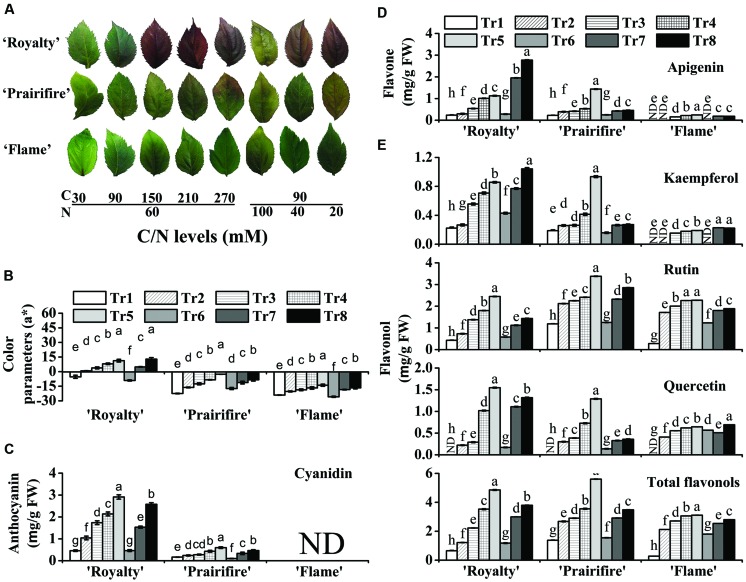
**High C/N ratios (both high C/normal N and normal C/high N) increase the accumulation of anthocyanins, flavones, and flavonols in leaves of the crabapple *Malus* sp. cultivars ‘Royalty,’ ‘Prairifire,’ and ‘Flame.’ (A)** Typical leaf phenotypes of ‘Royalty,’ ‘Prairifire,’ and ‘Flame’ grown under various C/N conditions. **(B)** Color parameters a* in leaves of ‘Royalty,’ ‘Prairifire,’ and ‘Flame’ grown under various C/N conditions. Contents of anthocyanins **(C)**, flavones **(D)**, and flavonols **(E)** in leaves of ‘Royalty,’ ‘Prairifire,’ and ‘Flame’ grown under various C/N conditions, measured by high pressure liquid chromatography (HPLC). The only detectable anthocyanin and flavone were cyanidin and apigenin, respectively. Three flavonols were detected: kaempferol, rutin, and quercetin. The sum of the three components represented total flavonol content. Tr1–Tr8 correspond to 30C/60N, 90C/60N, 150C/60N, 210C/60N, 270C/60N, 90C/100N, 90C/40N, and 90C/20N, respectively. Error bars correspond to the SEM ± SE of three replicate analyses. Different letters above the bars indicate significantly different values (*P* < 0.05) calculated using one-way ANOVA followed by a Duncan’s multiple range test.

The anthocyanin pigmentation phenotypes of ‘Royalty,’ ‘Prairifire,’ and ‘Flame’ under various C/N conditions are shown in **Figure 2A**. When plantlets of the ever-red cultivar ‘Royalty’ were cultured on media containing high C/normal N (150C/60N, 210C/60N, or 270C/60N) or normal C/low N (90C/40N or 90C/20N), anthocyanin pigmentation was observed, in the form of red pigmentation visible in the leaves (**Figure 2A**). Similarly, plantlets of the spring-red leaf cultivar ‘Prairifire’ grown under high C/normal N (150C/60N, 210C/60N, or 270C/60N) or normal C/low N (90C/40N or 90C/20N) conditions displayed a faint-red color at the leaf tips or along the leaf margins (**Figure 2A**). The pigmentation intensity of ‘Royalty’ and ‘Prairifire’ both increased as C increased or N decreased, whereas the red pigmentation of ‘Royalty’ was always darker than that of ‘Prairifire’ under all C/N conditions. In contrast, plantlets of the ever-green leaf cultivar ‘Flame’ did not develop any anthocyanin pigmentation under any of the C/N regimes, and remained different degrees of green (**Figure 2A**).

Plantlets under various C/N conditions displayed significant differences in color parameters and anthocyanin content (**Figure 2B** and Supplementary Table S1). Hunter a* increased with an increase in C or a decrease in N in all tested samples, particularly in ‘Royalty’ and ‘Prairifire,’ where it increased substantially as foliage color changed from green to red (**Figure 2B**). This was consistent with the variation in anthocyanin levels in these two cultivars when grown under different C/N conditions. When N was kept constant (60 mM), anthocyanin levels in ‘Royalty’ ranged from 0.46 mg g^-1^ FW under 30C to 2.91 mg g^-1^ FW under 270C. When N decreased from 100 to 20 mM at constant C levels (90 mM), anthocyanin levels in ‘Royalty’ ranged from 0.46 to 2.57 mg g^-1^ FW (**Figure 2C**). In contrast, lower amounts of anthocyanins were detected in ‘Prairifire’ under all C/N regimes, ranging from 0.1 to 0.6 mg g^-1^ FW (**Figure 2C**). Anthocyanins were not detected in ‘Flame’ under any C/N conditions (**Figure 2C**).

### High C/N Induces Anthocyanin Accumulation in Calli

**FIGURE 3:**
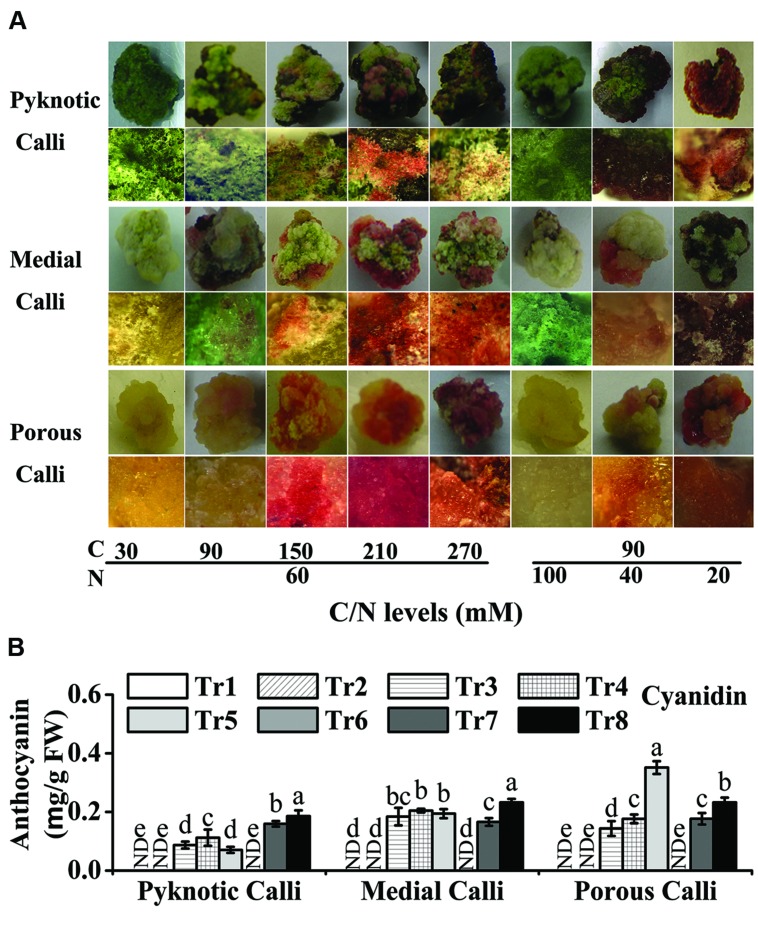
**High C/N ratios (both high C/normal N and normal C/high N) increase the accumulation of anthocyanins in calli of crabapple cultivar ‘Spring Snow’. (A)** Phenotypes of pyknotic, medial, and porous calli cultured under various C/N conditions. The images in the second, fourth and sixth lines are magnified views. **(B)** Anthocyanin levels in pyknotic, medial, and porous calli cultured under various C/N conditions. Tr1–Tr8 correspond to 30C/60N, 90C/60N, 150C/60N, 210C/60N, 270C/60N, 90C/100N, 90C/40N, and 90C/20N, respectively. Error bars correspond to the SEM ± SE of three replicate analyses. Different letters above the bars indicate significantly different values (*P* < 0.05) calculated using one-way ANOVA followed by a Duncan’s multiple range test.

Three types of crabapple calli all showed much more intense anthocyanin pigmentation when cultures under high C/N ratios than under other C/N ratios. When N was kept constant (60 mM), increasing C resulted in an increased red pigmentation in pyknotic calli, medial calli, and porous calli. For example, 270C induced the most intense pigmentation in pyknotic calli, followed by 210C, 150C, 90C, and finally 30C (**Figure 3A**). The same trend of pigmentation patterns was observed in medial calli and porous calli cultured at five different C levels. In addition, the effects of decreasing N on anthocyanin pigmentation were similar to those that resulted from increasing C (**Figure 3A**).

In the eight C/N conditions tested, the levels of anthocyanins ranged from 0.00 to 0.19 mg/g FW (FW) in pyknotic calli, 0.00 to 0.23 mg/g FW in medial calli, and 0.00 to 0.35 mg/g FW in porous calli (**Figure 3B**). The anthocyanin levels of pyknotic calli, medial calli, and porous calli were highest under 90C/20N, 90C/20N, and 270C/60N conditions, respectively (**Figure 3B**).

### High C/N Increases Flavone and Flavonol Accumulation in Plantlets

Flavone and flavonol levels in extracts from the leaves and calli were measured by HPLC; however, they were not detected in all three types of calli grown under any of the tested C/N conditions. The levels of flavones and flavonols in leaves increased as C increased or N decreased, which was similar to the trend observed for anthocyanins. Apigenin was the only detectable flavone in the leaves, and its content increased to different degrees in ‘Royalty,’ ‘Prairifire,’ and ‘Flame’ with increasing C levels, or decreasing N levels (**Figure 2D**). In comparison, the concentration of apigenin was lower in the leaves of ‘Flame,’ and changed less than that in ‘Royalty’ and ‘Prairifire’ in response to the different C/N ratios (**Figure 2D**).

Three flavonols, kaempferol, rutin, and quercetin, were detected in the crabapple leaves, and the levels of all three, as well as the total flavonol content, all increased as C increased or N decreased, with the exceptions of kaempferol in ‘Prairifire’ and ‘Flame’ and quercetin in ‘Flame’ under decreasing N content. Notably, there were not any detectable quercetin in the leaves of three crabapple cultivars grown under 30C/60N conditions, indicating that the synthesis of quercetin is sensitive to the C/N ratio. Finally, the total flavonol levels in the leaves of ‘Royalty’ and ‘Prairifire’ were substantially higher than those in the leaves of ‘Flame’ (**Figure 2E**).

### Carbon and Nitrogen Balance Regulates the Expression of Genes Related to Flavonoid Biosynthesis

To elucidate the molecular mechanisms involved in the regulation by the C/N conditions of flavonoid metabolism, the transcript abundance of flavonoid-related genes was examined by qRT-PCR, the primers and product sizes of which are shown in **Table 3**.

**FIGURE 4:**
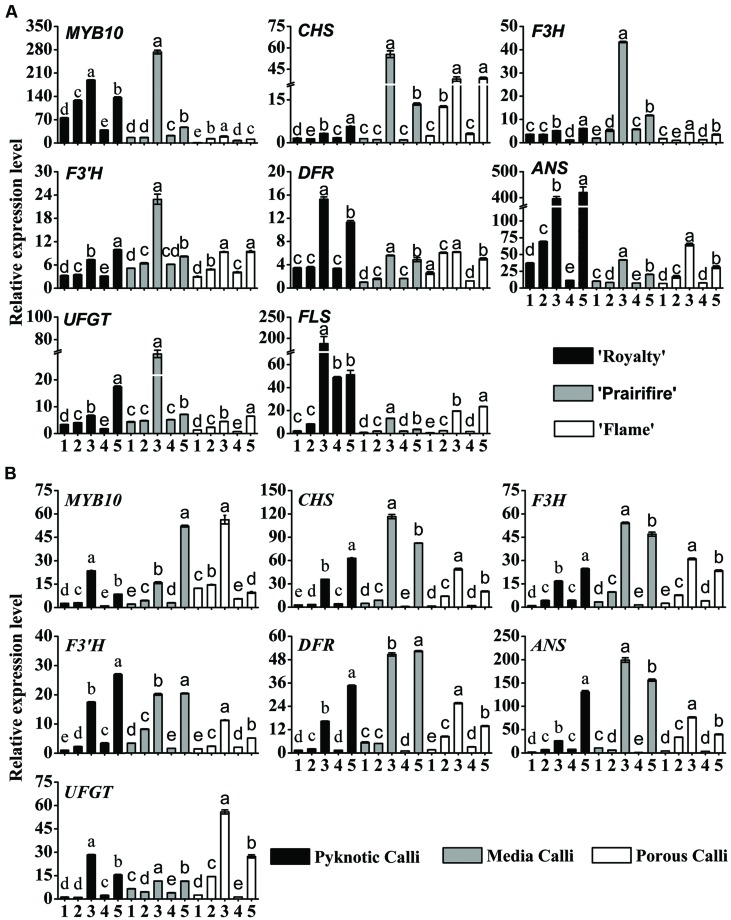
**Relative expression profiles of flavonoid/anthocyanin biosynthetic and regulatory genes in leaves **(A)** and calli **(B)** of crabapple *Malus* sp. cultivars grown under various C/N conditions**. Real-time PCR was used to analyze the expression levels of *MYB10*, *CHS*, *F3H*, *F3’H*, *DFR*, *ANS*, *UFGT*, and *FLS*. All real time-PCR reactions were normalized using the Ct value corresponding to a *Malus* crabapple 18S ribosomal RNA gene (DQ341382). Samples referred to on the *x* axis: (1) 30C/60N, (2) 90C/60N, (3) 150C/60N, (4) 90C/100N, and (5) 90C/20N. Error bars correspond to the SEM ± SE of three replicate analyses. Different letters above the bars indicate significantly different values (*P* < 0.05) calculated using one-way ANOVA followed by a Duncan’s multiple range test.

In the leaves of three cultivars, *MYB10* expression levels were substantially increased by high C/N ratios (150C/60N or 90C/20N), an increase that applied to all the genes tested, from *CHS* through to *UFGT* and *FLS* (**Figure 4A**), suggesting a coordinated up-regulation of the entire biosynthetic pathway. Under 150C/60N conditions, a much higher fold transcription change of *CHS*, *F3H*, *F3′H*, *UFGT* was observed for the spring-red leaf cultivar ‘Prairifire’ compared to the ever-red leaf cultivar ‘Royalty’ and the ever-green leaf cultivar ‘Flame’ (**Figure 4A**). The expression levels of *FLS* exhibited a lower fold increase in ‘Prairifire’ than in ‘Royalty’ and ‘Flame.’ The expression levels of *ANS* and *UFGT* increased most in leaves under high C/N conditions (150C/60N or 90C/20N), matching the observed accumulation of anthocyanin and the pigmentation phenotype of ‘Royalty’ and ‘Prairifire.’ However, transcript levels of genes in the early part of the flavonoid synthesis pathway (*CHS*, *F3H*, and *F3′H*) were lower in the ever-red leaf cultivar ‘Royalty’ than in the spring-red leaf cultivar ‘Prairifire.’

In three types of calli, the expression patterns of *MYB10* and six structural genes (*CHS*, *F3H*, *F3′H*, *DFR*, *ANS*, *UFGT*) were similar to those in the leaves. All tested samples showed much higher transcript levels under high C/N conditions (150C/60N or 90C/20N) than under low C/N conditions (30C/60N, 90C/60N, or 90C/100N) (**Figure 4B**). *FLS* expression was not detected in calli grown under all tested C/N conditions. These results suggest that the expression levels of flavonoid pathway biosynthetic genes were consistent with the accumulation of anthocyanins, flavones, and flavonols in crabapple leaves and calli under various C/N conditions.

### Correlation Analysis between C/N Regimes, Flavonoid Content, and Related Gene Expression

**FIGURE 5:**
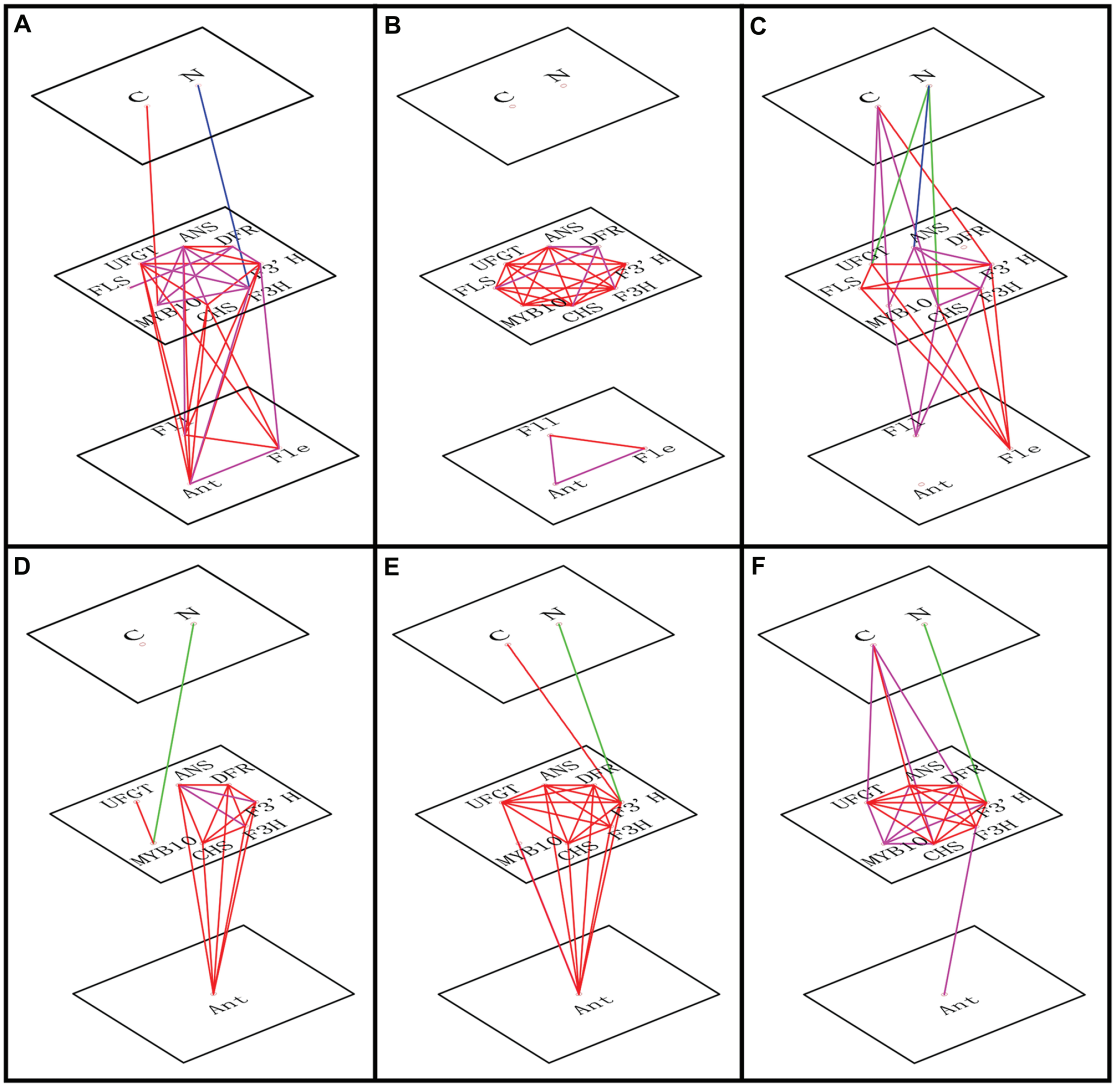
**Correlations of transcript levels among genes, between genes and C or N levels and between genes and flavonoid content (anthocyanin, Ant; flavone, Fle; and flavonol, Fll).** The red line indicates a positive correlation (Pearson correlation coefficient, *P* < 0.01), the magenta line indicates a positive correlation (Pearson correlation coefficient, *P* < 0.05), the blue line shows negative correlation (Pearson correlation coefficient, *P* < 0.01), and the green line shows negative correlation (Pearson correlation coefficient, *P* < 0.05). The relationships are shown for leaves of ‘Royalty’ **(A)**, ‘Prairifire’ **(B)**, and ‘Flame’ **(C)** cultivars, and for calli of crabapple *Malus* sp.; specifically pyknotic **(D)**, medial **(E)**, and porous **(F)** calli.

To identify which factors significantly influenced anthocyanin accumulation in response to the varying C or N conditions, a correlation analysis was conducted incorporating flavonoid contents, expression levels of relative genes and the variable C/N conditions (**Figure 5**). We determined that C content was positively related to the expression levels of *MYB10* in the leaves of ‘Royalty’ and ‘Flame’; however, this relationship was not significant in the leaves of ‘Prairifire’ and the calli of ‘Spring Snow.’ Meanwhile, N had a significant negative correlation with the expression levels of both regulatory and structural genes in all tested samples, except in the leaves of ‘Prairifire.’ Consistently, when the influence of C/N regimes became significant, stronger correlations emerged between the genes and flavonoid content (**Figures 5A–F**).

## Discussion

The balance between C and N must be tightly coordinated so that plants can optimize their growth and development (Coruzzi and Bush, 2001; Coruzzi and Zhou, 2001; Nunes-Nesi et al., 2010). Despite the fact that both C and N have been shown to influence flavonoid accumulation in different plant species and in specific organs/tissues (Stewart et al., 2001; Hara et al., 2003; Lea et al., 2007; Ram et al., 2011), the interacting effects of C and N on flavonoid synthesis remain unclear. Given the increasing concentration of atmospheric CO_2_ (Takatani et al., 2014), and N pollution in the environment (Camargo and Alonso, 2006), there is increasing interest in the role of the C/N balance on plant growth, development and metabolism (Nunes-Nesi et al., 2010; Carlisle et al., 2012; Fulweiler et al., 2014).

In this study, under normal N concentrations, increasing C content from 30 to 270 mM led to several phenotypic, physiological, and metabolic changes in the crabapple plantlets. Plantlet morphology was significantly altered, with an increase in FW and proliferation rate and a reduction in height (**Figure 1**). The changes were accompanied by increased levels of anthocyanins, flavones, and flavonols, as well as the expression of the corresponding biosynthetic genes in the leaves. Shoot cultures of *Fagus sylvatica* and *F. orientalis* grown on high glucose concentrations have previously shown analogous effects, with increased shoot multiplication (Cuenca and Vieitez, 2000). Similarly, Jo et al. (2009) found that a sucrose concentration in the growth medium of >3% increased the proliferation, FW, and dry weight of *Alocasia amazonica* plantlets. These results demonstrate that an increase in the C supply can lead to an increase in the proliferation rate. However, an excessively high concentration of C may induce osmotic stress and thus decrease the survival of the plantlets (Ncube et al., 2014). Results also show that, under normal C concentration, the N concentration which is too high or too low is not conductive to the development of crabapple, so proper control of N concentration is very important for plants Additionally, the crabapple calli were evaluated to verify the interactive effects of C and N on plant growth. High C/N ratios (both high C/normal N and normal C/low N) inhibited calli growth to differing degrees, probably due to osmotic stress or nutrient deficiency, as previously suggested (Mathur et al., 2010; Ram et al., 2011).

Increased pigmentation was observed in both leaves and calli of the different genotypes grown on high C/N media. This pigmentation was obvious in the leaves of the ever-red cultivar ‘Royalty’ and the spring-red cultivar ‘Prairifire,’ as well as in the three calli types (**Figures 2A and 3A**). These changes all coincided with an increase in anthocyanin content (**Figures 2C and 3B**). Our results are in agreement with previous studies demonstrating that anthocyanin biosynthesis in *A. thaliana* seedlings is strongly induced by high ratios of C/N (Martin et al., 2002; Gao et al., 2008; Sato et al., 2009; Zheng, 2009). Interestingly, no visible pigmentation or detectable anthocyanin was observed in the non-colored-leaves of ‘Flame’ grown under high C/N conditions, which differed from the results obtained with the colored leaves of ‘Royalty’ and ‘Prairifire,’ and in the calli of the non-colored leaf cultivar ‘Spring Snow’ (**Figures 2** and **3**). These results indicate that high C/N ratios are not sufficient to induce anthocyanin biosynthesis in differentiated tissues of green genotypes. It was reported that cabbage (*Brassica oleracea* var. *capitata* L.) grown under nutrient stress showed similar effects, with the anthocyanin content increasing greatly in red cultivars but only slightly in green cultivars (Yuan et al., 2009).

The leaves of crabapple were found to contain flavones and flavonols, which appeared to show a coordinated biosynthetic pathway response, with an increase in the concentrations of apigenin, kaempferol, rutin, and quercetin in response to high C/N ratios in all three cultivars. These results are consistent with previous studies showing the accumulation of quercetin and kaempferol in *A. thaliana* and tomato leaves after N withdrawal (Lea et al., 2007; Stewart et al., 2001). However, in three types of calli, flavones and flavonols were not detected. We speculate that cell dedifferentiation in our tissue culture might result in a reduction in the formation of flavone and flavonol.

In addition to flavonoid content, the expression of flavonoid biosynthetic genes was coordinately up-regulated by high C/N treatments. The expression levels of general flavonoid pathway genes (from *CHS* through to *UFGT*), as well as the specific *FLS* gene were substantially increased by high C/N ratios in the leaves of the three cultivars, matching the modest accumulation of anthocyanins, flavones, and flavonols, and also the color phenotype (**Figure 2**). The expression of the general flavonoid pathway genes was enhanced in three types of calli, which was congruent with the accumulation of anthocyanin (**Figure 3**). These results are also consistent with previous studies using *A. thaliana* seedlings, where anthocyanin accumulation in response to high C/N ratios was found to result from the up-regulation of *CHS* expression (Martin et al., 2002; Sato et al., 2009). Even though changes in *CHS* transcript abundance in leaves under high C/N ratios showed the same trends between the three cultivars, their expression levels were significantly lower in the ever-red leave cultivar ‘Royalty’ than in the other two cultivars. And as our previous studies showed the transcript levels of *CHS* were not always higher in the red leaves than in the green leaves (Zhang et al., 2014; Tian et al., 2015). The lack of correlation between RNA levels and phenotype is indicative of post-transcriptional control, post-translational control, or any other influences (Gou et al., 2011). Interestingly, we found that the expression levels of the late anthocyanin biosynthetic genes, *ANS* and *UFGT* in particular, were consistent with the concentration of anthocyanin in the leaves of the three cultivars grown under different C/N conditions. This corroborates the results of Zhang et al. (2014), who found that the expression levels of *ANS* and *UFGT* in crabapple leaves were up-regulated by a low pH treatment. Similarly, in this study, the expression levels of *FLS* were clearly enhanced by high C/N ratios, matching the accumulation of all three flavonol compounds. However, *FLS* expression was not detected in all three types of calli under all tested C/N ratios in this study, which may explain the absence of flavonols. In summary, these results indicate that the multiple late pathway genes determine the differential accumulation of anthocyanins, flavones, and flavonols.

Transcriptional regulation of flavonoid structural genes by regulatory genes provides an additional level of control. Numerous studies have demonstrated that flavonoid biosynthesis is regulated by a complex of MYB TFs, basic helix-loop-helix (bHLH) TFs, and WD-repeat proteins (Baudry et al., 2004). In this regard, particular attention has hitherto been devoted to MYB10 (Espley et al., 2007; Wang et al., 2010; Telias et al., 2011; Wang et al., 2011; Jiang et al., 2014; Tian et al., 2015). Under high C/N conditions, in both crabapple leaves and calli, the transcript levels of *MYB10* were up-regulated, which is consistent with the transcript levels of the flavonoid structural genes and the red phenotype. However, further research is necessary to elucidate how *MYB10* interacts with bHLH, and WD40 proteins to regulate the genes in the flavonoid biosynthetic pathway under varied C/N conditions.

Recently, over-application of synthetic N fertilizers has become a common problem in intensively farmed regions of China (Ma et al., 1999; Miao et al., 2010). It has been reported that high levels of N in cereal, ornamental and fruit crops, and trees are associated with a reduction in the accumulation of anthocyanins and, consequently, in the production of economically important products with esthetically pleasing colors and nutrition value (Marsh et al., 1996; Keller et al., 1999; Wargo et al., 2003; Nava et al., 2007; Camargo and Alonso, 2006). In addition, elevated CO_2_ levels can modulate plant growth, development and metabolism, including flavonoid/anthocyanin biosynthesis (Tallis et al., 2010; Hachiya et al., 2014; Takatani et al., 2014; Van Groenigen et al., 2014). The results described in this study provide the basis for improving our understanding of the significance of the C/N balance on flavonoid metabolism at the molecular level in controlled environments. However, more research should be conducted to evaluate the influence of N nutrition under elevated CO_2_ conditions on flavonoid metabolism.

## Conflict of Interest Statement

The authors declare that the research was conducted in the absence of any commercial or financial relationships that could be construed as a potential conflict of interest.

## Abbreviations

ANS: anthocyanidin synthase
6-BA: 6-benzylaminopurine
C: carbon
CHI: chalcone isomerase
CHS: chalcone synthase
2,4-D: 2,4- dichlorophenoxyacetic acid
DFR: dihydroflavonol 4-reductase
F3H: flavanone 3β-hydroxylase
F3′H: flavonoid 3’-monooxygenase
UFGT: uridine diphosphate (UDP)-glucose: flavonoid 3-*O*-glycosyltransferase
FLS: flavonol synthase
FW: fresh weight
MS: Murashige and Skoog medium
N: nitrogen
NAA: α-naphthaleneacetic acid
TF: transcription factor.
