# Supplementary material for: Promotion of flavonoid biosynthesis in leaves and calli of ornamental crabapple (Malus sp.) by high carbon to nitrogen ratios
Source: Front Plant Sci. 2015 Sep 1;6:673. doi: 10.3389/fpls.2015.00673 (PMC4555657; doi:10.3389/fpls.2015.00673)
Supplement: Supplementary file 1 [file Table_1.DOC]

**Table S1. Color parameters (L*, b*) and hue angle (h*) of leaves from crabapple *Malus* spp. cultivars ‘Royalty’, ‘Prairifire’ and ‘Flame’**

| Treatment | L | | |  | b | | |  | H | | |
| --- | --- | --- | --- | --- | --- | --- | --- | --- | --- | --- | --- |
|  | Royalty | Prairifire | Flame |  | Royalty | Prairifire | Flame |  | Royalty | Prairifire | Flame |
| 30C/60N | 39.36±7.89ab | 44.46±2.11c | 48.30±3.53ab |  | 20.42±7.83ab | 33.10±1.76ab | 33.81±2.41b |  | 105.09±1.72b | 124.26±1.39a | 125.56±2.15ab |
| 90C/60N | 39.13±8.44ab | 49.31±2.04bc | 47.07±4.15ab |  | 15.48±3.46abc | 26.30±1.01bc | 27.24±1.57c |  | 86.94±1.18c | 121.88±0.43a | 126.81±1.60a |
| 150C/60N | 32.56±3.41b | 44.11±6.46c | 49.69±6.84ab |  | 8.87±1.28c | 21.18±1.74c | 26.57±1.44cd |  | 66.94±4.75d | 120.64±1.51a | 125.12±2.32ab |
| 210C/60N | 31.69±2.28b | 48.72±0.91bc | 51.73±8.40ab |  | 9.73±1.66c | 21.45±3.46c | 21.99±1.75e |  | 50.16±3.12e | 110.19±4.43b | 127.38±0.16a |
| 270C/60N | 31.55±0.59b | 64.42±5.89a | 57.23±0.64a |  | 9.34±0.68c | 12.01±3.78d | 18.37±1.69e |  | 39.77±3.07f | 102.22±5.19c | 127.31±0.59a |
| 90C/100N | 45.85±2.86a | 53.13±1.33b | 49.39±3.32ab |  | 22.13±5.78a | 32.31±6.48ab | 41.91±1.96a |  | 113.20±6.07a | 118.68±4.99a | 121.70±1.73b |
| 90C/40N | 33.77±4.11b | 49.25±6.21bc | 45.46±11.45ab |  | 12.58±4.10bc | 20.01±5.93c | 22.70±3.63de |  | 67.69±6.28d | 120.26±7.02a | 129.40±4.93a |
| 90C/20N | 41.26±7.90ab | 60.87±1.49a | 42.32±9.94b |  | 15.36±4.19abc | 36.24±6.63a | 19.94±3.00e |  | 49.63±4.23e | 102.76±1.32c | 130.10±2.96a |
